# Response of plasma microRNAs to nusinersen treatment in patients with SMA

**DOI:** 10.1002/acn3.51579

**Published:** 2022-05-18

**Authors:** Irina T. Zaharieva, Mariacristina Scoto, Karolina Aragon‐Gawinska, Deborah Ridout, Bruno Doreste, Laurent Servais, Francesco Muntoni, Haiyan Zhou

**Affiliations:** ^1^ Developmental Neurosciences Research and Teaching Department, Dubowitz Neuromuscular Centre Great Ormond Street Institute of Child Health, University College London London UK; ^2^ Great Ormond Street Hospital London UK; ^3^ Institute I‐Motion Hôpital Armand Trousseau Paris France; ^4^ Neurology Department Medical University of Warsaw Warsaw Poland; ^5^ Population, Policy & Practice Department UCL Great Ormond Street Institute of Child Health London UK; ^6^ NIHR Great Ormond Street Hospital Biomedical Research Centre London UK; ^7^ Department of Paediatrics, MDUK Oxford Neuromuscular Centre University of Oxford Oxford UK; ^8^ Department of Paediatrics, Neuromuscular Reference Center Centre Hospitalier Universitaire de Liège Liège Belgium; ^9^ Genetics and Genomic Medicine Research and Teaching Department Great Ormond Street Institute of Child Health, University College London London UK

## Abstract

**Objective:**

Spinal muscular atrophy (SMA) is a common genetic cause of infant mortality. Nusinersen treatment ameliorates the clinical outcome of SMA, however, some patients respond well, while others have limited response. We investigated microRNAs in blood samples from SMA patients and their response to nusinersen treatment evaluating the potential of circulating microRNAs as biomarkers for SMA.

**Methods:**

In a discovery cohort study, microRNA next‐generation sequencing was performed in blood samples from SMA patients (SMA type 2, *n* = 10; SMA type 3, *n* = 10) and controls (*n* = 7). The dysregulated microRNAs were further analysed in the therapeutic response cohort comprised of SMA type 1 patients (*n* = 22) who had received nusinersen treatment, at three time points along the treatment course (baseline, 2 and 6 months of treatment). The levels of the studied microRNAs were correlated to the SMA clinical outcome measures.

**Results:**

In the discovery cohort, 69 microRNAs were dysregulated between SMA patients and controls. In the therapeutic response cohort, the baseline plasma levels of miR‐107, miR‐142‐5p, miR‐335‐5p, miR‐423‐3p, miR‐660‐5p, miR‐378a‐3p and miR‐23a‐3p were associated with the 2 and 6 months response to nusinersen treatment. Furthermore, the levels of miR‐107, miR‐142‐5p, miR‐335‐5p, miR‐423‐3p, miR‐660‐5p and miR‐378‐3p at 2 months of treatment were associated with the response after 6 months of nusinersen treatment.

**Interpretation:**

Blood microRNAs could be used as biomarkers to indicate SMA patients’ response to nusinersen and to monitor the efficacy of the therapeutic intervention. In addition, some of these microRNAs provide insight into processes involved in SMA that could be exploited as novel therapeutic targets.

## Introduction

Spinal muscular atrophy (SMA) is an autosomal recessive neuromuscular disorder, characterised by progressive muscle atrophy and paralysis, resulting from motor neuron degeneration in the spinal cord.^1^ SMA is caused by bi‐allelic loss of function of the *survival motor neuron 1 gene* (*SMN1*).^2^ Its paralogous neighbouring centromeric gene, *SMN2*, is intact in all patients but contains a single nucleotide variation in exon 7. The variation affects a splice enhancer and determines the exclusion of exon 7 in the majority of its transcripts, leading to an unstable SMN protein that cannot substitute the function of full‐length *SMN1*.^3^, ^4^

Nusinersen is an intrathecally administered antisense oligonucleotide drug and is the first approved disease‐modifying treatment for SMA. It increases full‐length SMN protein by augmenting *SMN2* exon 7 splicing in the mature mRNA transcripts.^5^, ^6^, ^7^ While the extent of clinical response observed in SMA patients is unequivocal and life changing compared to the natural history of the disease, it is increasingly evident that patients respond differently to nusinersen treatment. Some affected children respond well to the acquisition of unexpected motor abilities, while others have a more limited clinical response.^5^ Variables, including disease severity and the interval between disease onset and the start of treatment, are reported to play a role, but they do not account for the entire spectrum of clinical responses.^8^, ^9^ It is therefore important to identify informative biomarkers which may be used to better understand the variables that determine the response to nusinersen treatment.

MicroRNAs are a class of small (~22 nt) endogenous non‐protein‐coding RNA molecules that post‐transcriptionally regulate gene expression. They are emerging biomarkers for diagnosis and prognosis of diseases, as well as treatment response, in a number of disease fields.^10^, ^11^ MicroRNAs are known to play important roles in the regulation of muscle and central nervous system development.^12^, ^13^, ^14^ Dysregulation of microRNAs has been extensively investigated in animal models and patients with different neuromuscular disorders such as Duchenne muscular dystrophy and SMA.^15^, ^16^, ^17^, ^18^ Furthermore, disrupting microRNA biogenesis pathways by deleting Dicer 1 from spinal motor neurons in mice caused an SMA‐like neurodegenerative phenotype with motor neuron dysfunction and death, indicating the close connection between microRNAs and motor neuron health.^19^

Specific microRNA signatures in biofluids can provide useful non‐invasive tools to monitor disease progression and response to therapeutic intervention. We have previously reported the potential of serum microRNAs as non‐invasive and informative biomarkers of disease progression and response to experimental antisense therapy in SMA transgenic mice.^20^ MiR‐9, miR‐206 and miR‐132 were differentially expressed in the spinal cord, skeletal muscle and serum samples in SMA mice. After a single‐dose therapeutic morpholino antisense oligomer treatment in SMA mice, the pathological miR‐132 levels in the spinal cord, muscle and serum reversed to the normal levels. In addition, altered levels of miR‐9 and miR‐132 were detected in serum samples from SMA patients.

To further assess the role of microRNAs as biomarkers in SMA, we here conducted a deep microRNA discovery profiling study using next‐generation sequencing (NGS) in blood samples from SMA patients. We identified a list of microRNAs with significantly differential expression between the SMA patient cohort and healthy controls. The microRNA signatures were further studied in nusinersen‐treated SMA patients to assess the effect that therapeutic intervention had on their profile. The molecular data were correlated with the longitudinal clinical response to therapy measured by motor function scores.

Our data reveals the differential expression of microRNAs in response to nusinersen treatment in individuals with SMA and underlines the potential application of selected circulating microRNAs as biomarkers in this condition.

## Methods

### Patient cohort

For the discovery cohort study, serum samples from SMA patients and healthy controls were obtained from the MRC Center for Neuromuscular Diseases Biobank London (REC reference number 06/Q0406/33, http://www.cnmd.ac.uk). A summary of the patients is presented in Table 1. All patients had confirmed genetic diagnosis of SMA with a homozygous genomic deletion in the *SMN1* gene and two or three copies of the *SMN2* gene.

**Table 1:** Clinical data of the patients included in the discovery cohort study.

Sample ID	SMA type	Age at sample collection (years)	Gender	HFMS
SMA‐1	2	4.7	Male	23
SMA‐2	2	8	Male	26
SMA‐3	2	8.1	Female	10
SMA‐4	2	9.8	Female	19
SMA‐5	2	9.9	Female	4
SMA‐6	2	9.9	Female	15
SMA‐7	2	10.9	Male	2
SMA‐8	2	15.1	Male	NA
SMA‐9	2	15.2	Male	NA
SMA‐10	2	16.1	Male	NA
SMA‐11	3	4.7	Female	54
SMA‐12	3	6	Female	26
SMA‐13	3	8	Female	49
SMA‐14	3	9.6	Male	31
SMA‐15	3	10.5	Female	32
SMA‐16	3	12.4	Male	NA
SMA‐17	3	13	Female	37
SMA‐18	3	13	Female	NA
SMA‐19	3	13.5	Female	NA
SMA‐20	3	17	Male	NA

Presented are the type of SMA, age at sample collection, gender and HFMS score of the patients (NA = not available). HFMS, Hammersmith Functional Motor Scale; SMA, spinal muscular atrophy.

For the therapeutic response cohort study, blood samples for plasma extraction were collected from SMA patients (*n* = 22) treated with nusinersen at the Institute I‐Motion, Hôpital Armand Trousseau, Paris, France and the CHR Citadelle Hospital, Liège, Belgium.^21^, ^22^ All patients had a diagnosis of SMA type 1 and two or three *SMN2* copies. The mean age of diagnosis of the patients was 3.6 months and the mean age of start of treatment was 65 months (median = 19 months; age range 1–420 months) (Table 2). The samples were collected before the first intrathecal injection of nusinersen on day 1 as a baseline assessment, at 2 and 6 months of treatment. At each visit, the patients completed physiotherapy assessment using Hammersmith Infant Neurological Examination Section 2 (HINE‐2) and Children’s Hospital of Philadelphia Infant Test of Neuromuscular Disorders (CHOP‐INTEND) clinical outcome assessment scales.

**Table 2:** Clinical data of the patients included in the therapeutic cohort study.

Patient ID	SMN2 copy number	Age of symptoms onset (months)	Age at first dose (months)	CHOP‐INTEND pre‐treatment	HINE pre‐treatment	CHOP‐INTEND 2 months of treatment	HINE 2 months of treatment	CHOP‐INTEND 6 months of treatment	HINE 6 months of treatment
PN1	3	NBS	1	58	3	62	6	NA	NA
PN2	3	5	32.6	37	1	39	6	35	4
PN3	3	3	10	16	1	NA	2	20	3
PN4	3	<6	396	15	4	NA	NA	25	5
PN5	2	3	123	9	1	14	1	15	1
PN6	3	4	16.6	25	1	32	2	36	3
PN7	2	3	19	11	1	22	1	23	1
PN8	NA	2	4.2	35	2	34	2	38	2
PN9	3	5	52.4	NA	5	NA	5	NA	6
PN10	2	2	28.2	NA	6	NA	8	NA	10
PN11	3	3	19.1	32	2	30	2	34	5
PN12	2	4	15.7	27	1	25	1	28	0
PN13	3	4	41.9	NA	6	NA	7	NA	7
PN14	2	1	3	24	2	35	4	46	5
PN15	3	5	12	40	2	40	2	51	11
PN16	3	Pre‐symtomatic	5	63	11	64	18	64	24
PN17	3	3	27.7	22	1	22	1	26	1
PN18	3	5	15	29	2	32	3	43	NA
PN19	NA	0.5	155	NA	NA	NA	NA	NA	NA
PN20	2	5	5.8	31	2	39	3	49	5
PN21	2	6	420	NA	NA	NA	NA	NA	9
PN22	2	2	30.3	35	4	40	7	41	8

Presented are the age of onset, age at the first dose and motor milestone scores according to CHOP‐INTEND and HINE assessments at baseline, 2 and 6 months of treatment. CHOP‐INTEND, Children’s Hospital of Philadelphia Infant Test of Neuromuscular Disorders; HINE, Hammersmith Infant Neurological Examination Section 2; NA, not available; NBS, newborn screening; SMN, survival motor neuron.

### Serum microRNA next‐generation sequencing

MicroRNAs were extracted from 500 μL of serum, and after microRNA library preparation, the libraries were sequenced on Illumina Nextseq500 with average of 10 million reads per sample, 50 nt singe‐end reads.

The counts of microRNA were normalised by applying the trimmed mean of M‐values method.^23^ The analysis between the different groups was performed using a pairwise comparison of the normalised microRNA counts. The *p* values were derived by an exact test on the negative binomial distribution and were corrected for multiple testing using the Benjamini‐Hochberg method.^24^ The Benjamini‐Hochberg method, or false discovery rate (FDR), is the expected ratio of false‐positive results to the total number of positive results. FDR is less stringent than Bonferroni correction and has a greater power of detecting positive results; however, it might lead to increased Type I errors.^25^

### miRCURY LNA PCR

Custom miRCURY microRNA panels were designed for the validation of the significant microRNAs from the NGS screening. MicroRNAs were extracted from 200 μL of plasma using miRNeasy Serum/Plasma Kit (cat no. 217184) and then converted to cDNA using an miRCURY LNA RT Kit (cat. no. 339340). After diluting the cDNA with RNase‐free water in the ratio of 1:20, the miRCURY SYBR Green PCR mix was prepared and 10 μL of the mix was dispensed in each well of the miRCURY microRNA plates (miRCURY SYBR Green PCR Kit, cat. nos. 339345, 339346 and 339347). The RT‐PCR was carried out on Applied Biosystems StepOnePlus System with the following conditions: initial PCR activation of 2 min at 95°C and 40 cycles of denaturation at 95°C for 10 sec and combined annealing/extension for 60 sec at 56°C. Inter‐plate calibration (IPC) was carried out for each plate by calculating a calibration factor as a difference between the average of the IPC replicates for each plate and the average of IPC values from all plates. Each plate was calibrated by subtracting the calibration factor from all *Ct* values in the plate. The data analysis of the calibrated *Ct* values was carried out using 2^−ΔΔCT^ method.

### Assessment of patients’ motor function

The CHOP‐INTEND^26^ and the HINE^27^ total scores were collected at baseline, 2 and 6 months after starting nusinersen treatment.

### Statistical analysis

Univariate linear regression analysis was carried out to explore the relationship between levels of microRNAs and response to nusinersen treatment. Mann–Whitney test for independent samples or Wilcoxon‐signed rank tests for the analysis of paired samples were used to analyse the change in the levels of microRNAs during treatment with nusinersen. The statistical analyses were performed using IBM SPSS Statistics V25 software and the *p* value of <0.05 was considered statistically significant. Graphs were created using IBM SPSS Statistics V25 software and Prism v8 GraphPad.

## Results

### Circulating microRNAs profiling in SMA patients by next‐generation sequencing

**Figure 1:**
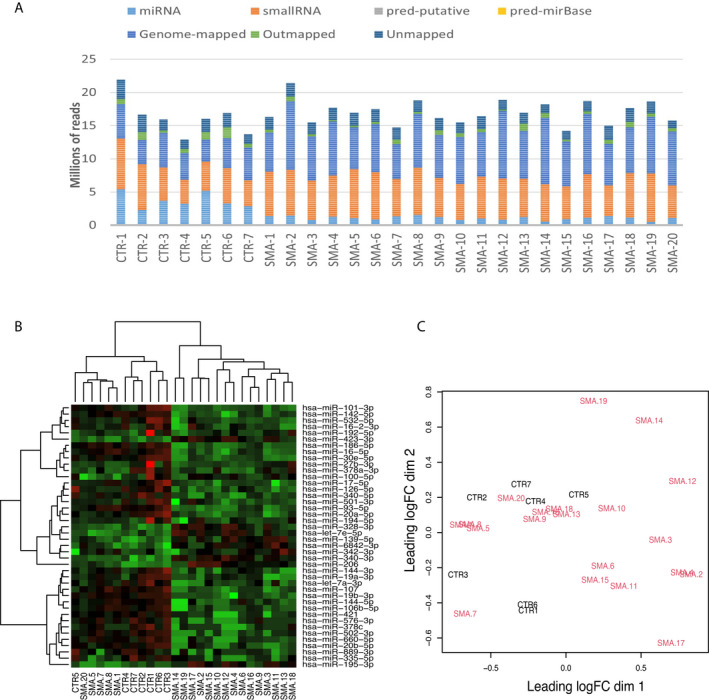
(A) Summary of the mapping of the reads for each sample. (B) Heatmap of microRNAs with different levels in serum in SMA and control samples. Rows correspond to the significant microRNAs and columns correspond to individual samples. Red represents an expression level above the mean, and green represents an expression level below the mean. (C) Multidimensional scaling plot showing the separation between the SMA and the control samples based on the expression profile. The SMA samples are presented in red, and the controls are in black. SMA, spinal muscular atrophy.

To identify circulating microRNAs differentially expressed between SMA patients and healthy controls, we carried out microRNA NGS in serum samples of SMA patients (type 2, *n* = 10; type 3, *n* = 10) and healthy controls (*n* = 7). On average 16.2 million reads were obtained from each sample. In the SMA patients, of the reads mapped to the human genome (hg19), on average 7% were mapped to microRNAs (mirBase release 20), and 36% were mapped to other small RNA sequences such as Y RNA, snRNA, snoRNA and tRNA. Forty‐four percent of the reads were mapped to locations in the genome where no microRNAs or small RNAs were located, 5% of the reads mapped to abundant sequences, for example polyA and polyC homopolymers or ribosomal RNA, and 12% of the reads failed to map to the reference genome. In the control samples, a higher number of reads mapped to microRNA sequences (23%) and а comparable number of reads mapped to small RNA sequences (36%), other genomic regions (28%), abundant sequences (5%) and 12% did not align to the reference genome (Fig. 1A).

We then set to explore the expression pattern of microRNAs between the SMA and the control samples by performing hierarchical clustering. To visualise the microRNAs with the highest variation between SMA and control samples, we normalised the data by converting the expression values to a log2 scale and the microRNAs with the highest coefficient of variation were selected for the analysis. Three main clades of the dendrogram were observed with the largest being formed of SMA samples, followed by a clade of control samples and a clade of SMA patients’ samples (Fig. 1B). Next, we analysed the separation of SMA and control samples and the multidimensional scaling plot illustrated the subtle difference in the expression profiles of SMA and control samples as they did not cluster separately (Fig. 1C).

The differentially enriched serum microRNAs were first compared between SMA patients and controls and then between SMA type 2 and type 3 patients. A total of 42 microRNAs were differentially expressed between SMA and controls (FDR <0.05). Of these, 14 microRNAs were upregulated and 28 were downregulated in SMA patients (Table S1). When the serum microRNAs detected in SMA type 2 were compared to the microRNAs in type 3 SMA patients, a total of 27 microRNAs were significantly different (uncorrected *p* < 0.05), however, they did not pass the threshold for multiple testing of FDR <0.05. Of these microRNAs, 16 were upregulated and 11 were downregulated in SMA type 2 compared to type 3 patients (Table S2).

For the subsequent analysis in the therapeutic response cohort study, we selected the 42 microRNAs differentially expressed between SMA patients and healthy controls, and in addition, the 27 microRNAs with different levels between SMA type 2 and type 3, even though they did not pass the significance threshold for multiple testing.

### Analysis of selected microRNAs in SMA patients treated with nusinersen

We next analysed the selected microRNAs in nusinersen‐treated SMA patients to explore the potential of these microRNAs to serve as biomarkers to monitor response to treatment.

Custom miRCURY LNA miRNA qPCR panels were designed for the analysis of the 69 microRNAs significant in the discovery study. Due to the limited availability of serum samples collected from nusinersen‐treated SMA patients, we carried out the screening in plasma samples. The plasma samples from nusinersen‐treated SMA patients were collected at three time points: pre‐treatment (baseline), 2 and 6 months of treatment.

MicroRNAs with a cycle threshold (Ct) value above 36 in more than 25% of the samples was considered as not reliably detected and were excluded from the analysis. This left us with 41 microRNAs for further statistical analysis. Data were normalised to the expression of hsa‐miR‐191‐5p which was detected as stably expressed across the different groups in the discovery study.^28^

### Prognostic microRNAs biomarkers in SMA type 1 patients treated with nusinersen

To understand the potential of these microRNAs as prognostic biomarkers for the response to therapeutic intervention, first, we analysed if the levels of microRNAs at baseline were associated with the response to nusinersen treatment assessed at 2 or 6 months of treatment. As a measure of the therapeutic response, we calculated the difference in CHOP‐INTEND or HINE scores at 2 or 6 months from the baseline score for each patient. A larger difference in the scores indicates a better response to treatment with a higher number of test items achieved in CHOP‐INTEND or HINE scales. Linear regression analysis between the baseline level of microRNAs and the change of scores in motor scales was then carried out.

**Figure 2:**
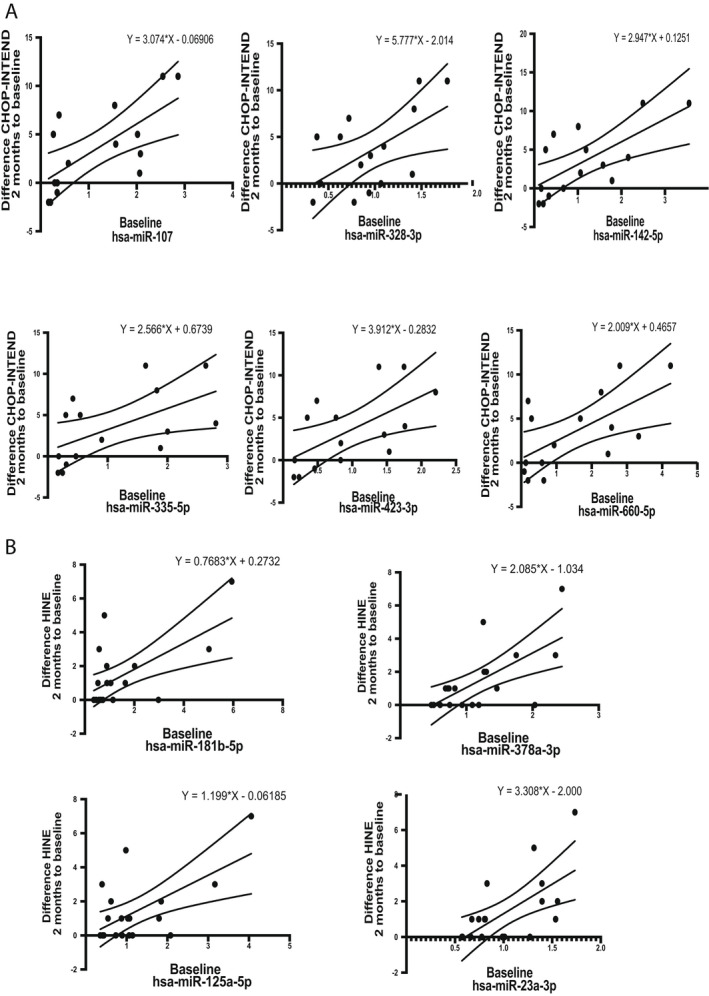
Linear regression relationship between the relative expression of the analysed microRNAs at baseline and the response to nusinersen treatment assessed using the difference between the CHOP‐INTEND (A) and HINE (B) scores at 2 months to the baseline score. Presented are the regression line with 95% confidence intervals of the best fit line and the regression equation. CHOP‐INTEND, Children’s Hospital of Philadelphia Infant Test of Neuromuscular Disorders; HINE, Hammersmith Infant Neurological Examination Section 2.

The baseline levels of six microRNAs (miR‐107, miR‐142‐5p, miR‐328‐3p, miR‐335‐5p, miR‐423‐3p and miR‐660‐5p) showed significant association with the response at 2 months of treatment measured using CHOP‐INTEND and four microRNAs (miR‐181b‐5p, miR‐378a‐3p, miR‐125a‐5p and miR‐23a‐3p) using HINE (Fig. 2 and Table 3).

**Table 3:** Linear regression analysis of baseline microRNAs relative expression to the functional improvement of the SMA patients at 2 months.

microRNA	*R* ^2^	Intercept	*β*	*p*‐value	Motor milestones scale
miR‐107	0.48	−0.07	3.07	0.004	CHOP‐INTEND
miR‐142‐5p	0.47	0.13	2.95	0.005	CHOP‐INTEND
miR‐328‐3p	0.33	−2.01	5.78	0.03	CHOP‐INTEND
miR‐335‐5p	0.31	0.67	2.57	0.03	CHOP‐INTEND
miR‐423‐3p	0.37	−0.28	3.91	0.02	CHOP‐INTEND
miR‐660‐5p	0.40	0.47	2.01	0.01	CHOP‐INTEND
miR‐181b‐5p	0.38	0.27	0.77	0.005	HINE
miR‐378a‐3p	0.42	−1.03	2.09	0.003	HINE
miR‐125a‐5p	0.38	−0.06	1.20	0.005	HINE
miR‐23a‐3p	0.39	−2.00	3.31	0.004	HINE

Presented are unstandardised coefficients, model *p* value and the motor milestone scale used in the analysis. CHOP‐INTEND, Children’s Hospital of Philadelphia Infant Test of Neuromuscular Disorders; HINE, Hammersmith Infant Neurological Examination Section 2; SMA, spinal muscular atrophy.

**Figure 3:**
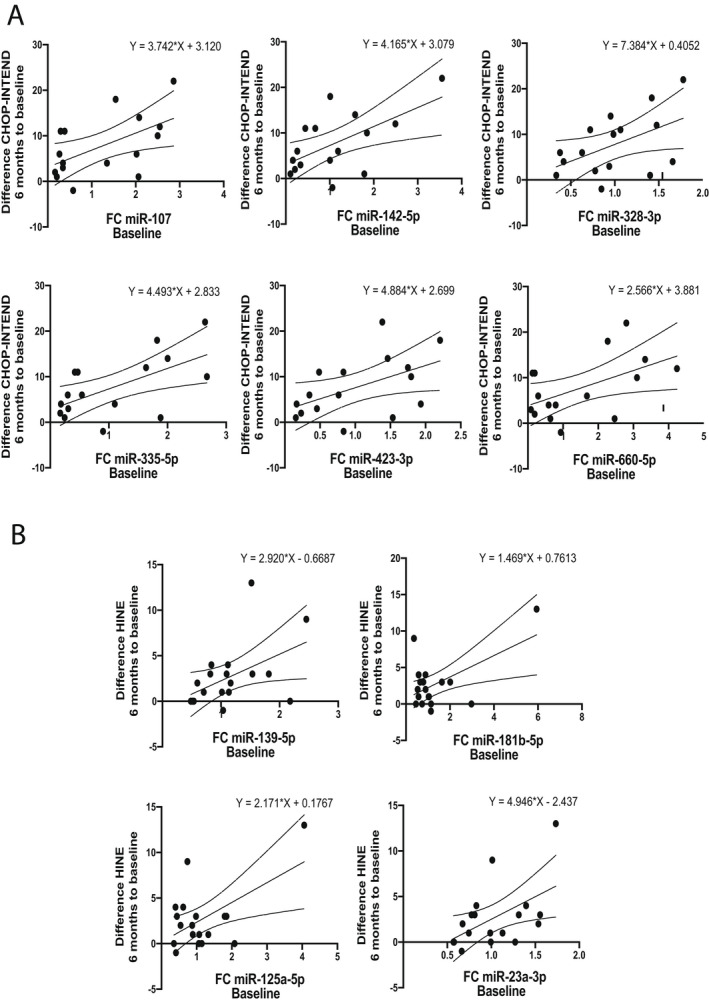
Linear regression relationship between the relative expression of the analysed microRNAs at baseline and the response to nusinersen treatment assessed using CHOP‐INTEND (A) and HINE (B) scores at 6 months to baseline score. Presented are the regression line with 95% confidence intervals of the best fit line and the regression equation. CHOP‐INTEND, Children’s Hospital of Philadelphia Infant Test of Neuromuscular Disorders; HINE, Hammersmith Infant Neurological Examination Section 2.

The baseline levels of the six microRNAs (miR‐107, miR‐142‐5p, miR‐328‐3p, miR‐335‐5p, miR‐423‐3p and miR‐660‐5p) also correlated to the response at 6 months assessed using CHOP‐INTEND, while miR‐181b, miR‐125a‐5p, miR‐23a‐3p and miR‐139‐5p showed significant association with the functional improvement at 6 months by HINE (Fig. 3 and Table 4).

**Table 4:** Linear regression analysis of baseline microRNAs relative expression to the functional improvement of the SMA patients at 6 months.

microRNA	*R* ^2^	Intercept	*β*	*p*‐value	Motor milestones scale
miR‐107	0.31	3.12	3.74	0.03	CHOP‐INTEND
miR‐142‐5p	0.36	3.08	4.17	0.01	CHOP‐INTEND
miR‐328‐3p	0.25	0.41	7.38	0.05	CHOP‐INTEND
miR‐335‐5p	0.36	2.83	4.49	0.01	CHOP‐INTEND
miR‐423‐3p	0.26	2.70	4.88	0.04	CHOP‐INTEND
miR‐660‐5p	0.27	3.88	2.57	0.04	CHOP‐INTEND
miR‐139‐5p	0.23	−0.67	2.92	0.04	HINE
miR‐181b‐5p	0.32	0.76	1.47	0.02	HINE
miR‐125a‐5p	0.32	0.18	2.17	0.02	HINE
miR‐23a‐3p	0.27	−2.44	4.95	0.03	HINE

Presented are unstandardised coefficients, model *p* value and the motor milestone scale used in the analysis. CHOP‐INTEND, Children’s Hospital of Philadelphia Infant Test of Neuromuscular Disorders; HINE, Hammersmith Infant Neurological Examination Section 2; SMA, spinal muscular atrophy.

**Figure 4:**
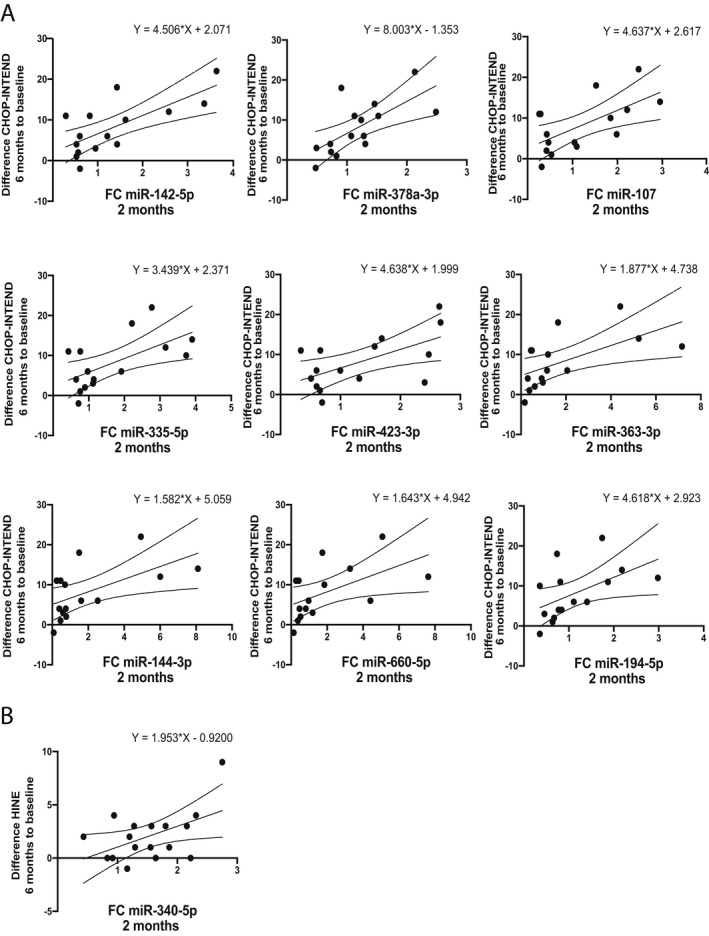
Linear regression relationship between the relative expression of the analysed microRNAs at 2 months and the response to nusinersen treatment assessed using CHOP‐INTEND (A) and HINE (B) scores at 6 months to the baseline score. Presented are the regression line with 95% confidence intervals of the best fit line and the regression equation. CHOP‐INTEND, Children’s Hospital of Philadelphia Infant Test of Neuromuscular Disorders; HINE, Hammersmith Infant Neurological Examination Section 2.

Further, we analysed if the relative expression of the studied microRNAs in plasma at 2 months of treatment correlated with the functional improvement of the patients after 6 months of treatment. When the relative expression of the microRNAs at 2 months and the change of CHOP‐INTEND scores at 6 months to baseline were analysed, nine microRNAs were correlated with the functional improvement of patients at 6 months of nusinersen treatment. MiR‐142‐5p (*R* ^2^ = 0.51, *p* = 0.003) and miR‐378a‐3p (*R* ^2^ = 0.46, *p* = 0.006) showed the highest prediction potential (Table 5). The linear regression plots are presented in Figure 4.

**Table 5:** Univariate linear regression analysis of the relative expression of microRNAs at 2 months and functional improvement of patients at 6 months of nusinersen treatment.

microRNA	*R* ^2^	Intercept	*β*	*p*‐value	Motor milestones scale
miR‐142‐5p	0.51	2.07	4.51	0.003	CHOP‐INTEND
miR‐378a‐3p	0.46	−1.35	8.00	0.006	CHOP‐INTEND
miR‐107	0.40	2.62	4.64	0.01	CHOP‐INTEND
miR‐335‐5p	0.38	2.37	3.44	0.02	CHOP‐INTEND
miR‐423‐3p	0.36	2.00	4.64	0.02	CHOP‐INTEND
miR‐363‐3p	0.35	4.74	1.88	0.02	CHOP‐INTEND
miR‐144‐3p	0.34	5.06	1.58	0.02	CHOP‐INTEND
miR‐660‐5p	0.30	4.94	1.64	0.04	CHOP‐INTEND
miR‐194‐5p	0.28	2.92	4.62	0.04	CHOP‐INTEND
miR‐340‐5p	0.26	−0.92	1.95	0.04	HINE

Presented are unstandardised coefficients, model *p* value and the motor milestones scale according to which the analysis was performed. CHOP‐INTEND, Children’s Hospital of Philadelphia Infant Test of Neuromuscular Disorders; HINE, Hammersmith Infant Neurological Examination Section 2.

The relative expression of miR‐340‐5p (*R* ^2^ = 0.255, *p* = 0.04) at 2 months was the only significant microRNA associated with the motor function improvement after 6 months of treatment assessed using HINE (Fig. 4 and Table 5).

In addition, we carried out correlation analysis between the baseline levels of the analysed microRNAs and the functional improvement of the patients after 2 and 6 months of treatment. The baseline level of miR‐335‐5p positively correlated with the functional improvement of patients at 2 months of treatment measured by CHOP‐INTEND (*r* = 0.59, *p* = 0.02) or HINE (*r* = 0.48, *p* = 0.03) and at 6 months assessed by CHOP‐INTEND (*r* = 0.5, *p* = 0.05) (Table S3). The baseline level of miR‐107 showed positive correlation with the functional improvement of the SMA patients at 2 and 6 months of treatment assessed by CHOP‐INTEND (*r* = 0.7, *p* = 0.004; *r* = 0.53, *p* = 0.03). MicroRNAs let‐7f‐5p (*r* = 0.57, *p* = 0.03), miR‐142‐5p (*r* = 0.66, *p* = 0.007), miR‐30e‐5p (*r* = 0.52, *p* = 0.05), miR‐340‐5p (*r* = 0.55, *p* = 0.03), miR‐423‐3p (*r* = 0.61, *p* = 0.01) and miR‐660‐5p (*r* = 0.6, *p* = 0.02) showed positive correlation with the improvement of the patients at 2 months assessed by CHOP‐INTEND (Table S3). The analysis also confirmed the positive correlation of the baseline level of miR‐23a‐3p with the functional improvement at 2 and 6 months treatment assessed by HINE (*r* = 0.58, *p* = 0.009; *r* = 0.54, *p* = 0.02). The positive correlation of the baseline levels of miR‐378a‐3p (*r* = 0.58, *p* = 0.009) with the improvement at 2 months treatment assessed by HINE was also validated. The analysis also identified baseline levels of miR‐19b‐3p (*r* = 0.46, *p* = 0.04) and miR‐139‐5p (*r* = 0.47, *p* = 0.05) to positively correlate with the HINE functional improvement of the patients at 2 and 6 months treatment respectively (Table S3).

We then set to examine if other factors such as the age of start of treatment and *SMN2* copy number could have any effect on the plasma levels of the microRNAs. At baseline, the age of the SMA patients ranged between 1 month and 35 years (mean age = 5 years; median age = 3.5 years) with the majority of patients being between 1 month and 1.6 years (12 patients), followed by six patients with age between 2.3 and 4.3 years and four patients above 10 years. Linear regression analysis between the age at the start of nusinersen treatment and the baseline levels of the most promising microRNAs (miR‐107, miR‐142‐5p, miR‐335‐5p, miR‐423‐3p, miR‐660‐5p, miR‐378a‐3p and miR‐23a‐3p) did not detect any significant positive association between the levels of microRNAs and the age of SMA patients except for miR‐107, which was higher in older patients (*R* ^2^ = 0.27, *p* = 0.01) (Fig. S1). This result indicates that while most of the microRNAs identified in this study are stable with age, the impact of age on the levels of miR‐107 should be considered when using miR‐107 as a biomarker in SMA. No significant difference was detected between the levels of the microRNAs and *SMN2* copy numbers.

### The response of plasma microRNA levels to nusinersen treatment

**Figure 5:**
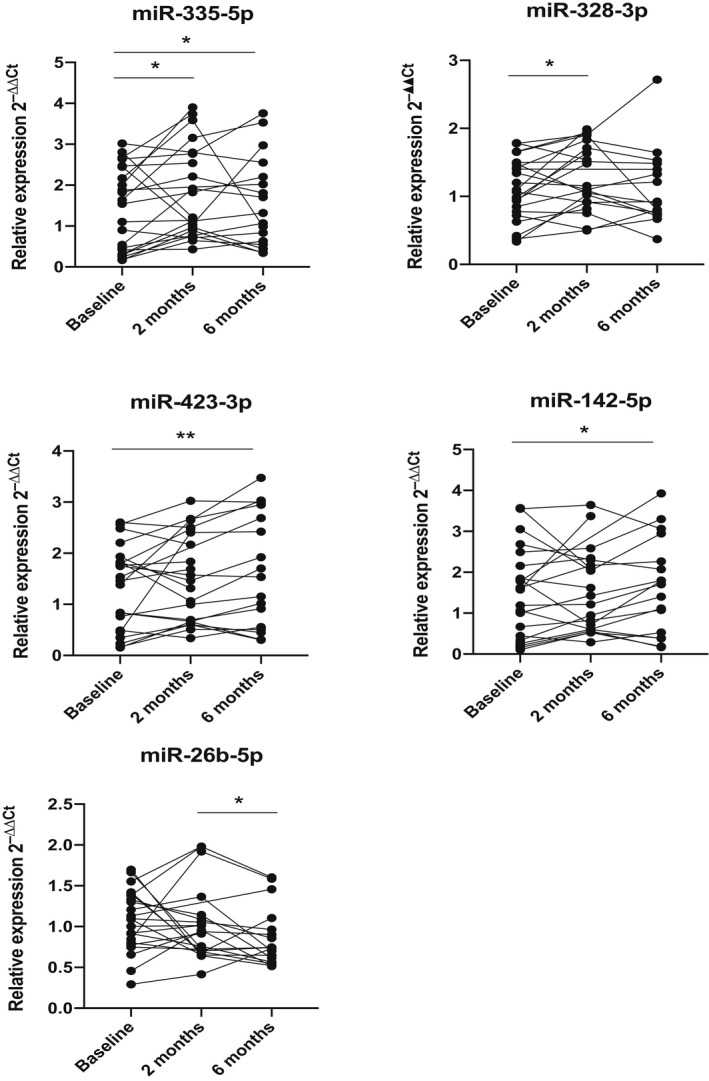
Relative expression of the analysed microRNAs in plasma samples from SMA type 1 patients at pre‐treatment, 2 and 6 months after nusinersen treatment. *p*values derived from the Wilcoxon matched‐pairs *t* test are presented with * and ** and correspond to *p* < 0.05 and *p* < 0.01 respectively. SMA, spinal muscular atrophy.

To determine whether the nusinersen treatment affected the plasma levels of the 41 microRNAs identified above, we carried out Wilcoxon matched‐pairs *t* test comparing the microRNAs plasma levels at baseline, 2 and 6 months of treatment. Increased levels at 2 months compared to the baseline levels were detected in miR‐335‐5p (*p* = 0.02) and miR‐328‐3p (*p* = 0.04) (Fig. 5). Increased plasma levels at 6 months of treatment compared to the baseline levels were detected, in addition to miR‐335‐5p (*p* = 0.02), also for miR‐423‐3p (*p* = 0.002) and miR‐142‐5p (*p* = 0.03) (Fig. 5). When the levels after 6 months of treatment were compared to the levels at 2 months, a significant difference was detected only for miR‐26b‐5p (*p* = 0.04) which showed a decrease of miR‐26b‐5p at 6 months of nusinersen treatment (Fig. 5).

The differences between the baseline, 2 and 6 months levels of miR‐335‐5p, miR‐328‐3p, miR‐423‐3p, miR‐142‐5p and miR‐26b‐5p were also analysed in the nusinersen‐treated SMA patients, grouped based on their clinical response into two categories: low responders and good responders. As a measure of response, we used the difference in the assessment score at 2 or 6 months of treatment relative to the baseline score. A difference in the CHOP‐INTEND score of at least 2 points or HINE score of at least 1 point was considered as a clinically significant improvement for the patients (good responders). A non‐parametric test (Mann–Whitney test) was carried out. We did not detect any significant difference in the levels of the five microRNAs between patients with the low response and good response.

Further, to determine if the identified changes in the levels of the five microRNAs were due to the age of the patients rather than an effect of the nusinersen treatment, we conducted a linear regression analysis between the age of the patients and the baseline plasma microRNA levels. No significant correlation was detected between the age of SMA patients and the baseline levels of miR‐335‐5p, miR‐328‐3p, miR‐423‐3p, miR‐142‐5p and miR‐26b‐5p.

## Discussion

Large heterogeneity in terms of clinical response to currently available treatments, ranging from the absence of response to impressive acquisition of motor milestones and survival, has been reported for all disease‐modifying therapies in SMA, with disease duration negatively correlated with patient response.^29^ Nevertheless, as other determinants of response are likely to exist, it is important to understand both the causes leading to the variable response as well as to identify biomarkers that could predict or monitor the response to treatment at an early stage.

Plasma levels of phosphorylated neurofilaments (NFs) have been previously used in SMA,^30^, ^31^ where elevated NF levels were detected in SMA type 1 patients compared to aged‐matched controls.^30^ The levels of NFs negatively correlated with the age of start of treatment and the severity of SMA patients. Moreover, nusinersen treatment was able to reduce NF levels, indicating that plasma NF levels could serve as biomarkers for disease activity and response to nusinersen treatment.^30^ Investigating the blood NF levels in older SMA patients (above 1 year old), however, demonstrated that NF declined with age to a level comparable to healthy controls,^30^ indicating the limited application of NFs as biomarkers in older SMA patients and pointing to the need of identifying additional biomarkers.

Muscle‐specific microRNAs (myomiRs) in the blood and CSF from SMA patients have recently been investigated as biomarkers to monitor patients’ response to nusinersen.^32^, ^33^ In the blood, miR‐133a reduction predicted patients’ response to nusinersen.^32^ In the CSF, lower miR‐206 and miR‐133 predicted a more robust clinical response to nusinersen in later‐onset SMA patients.^33^

Here we have conducted a high‐throughput microRNA NGS in blood samples of SMA patients and healthy controls to identify dysregulated microRNAs. Notably, a lower number of microRNAs were detected in SMA patients compared to controls (Fig. 1A). A hypothesis is that the SMN complex affects the microRNA biogenesis by interacting with a specific RNA‐binding protein^18^, ^34^, ^35^ and in the SMA context the deficiency of SMN may cause defects in microRNA biogenesis leading to a lower number of microRNAs in SMA patients.

Although at the time of conducting the study in nusinersen‐treated SMA 1 patients, a report demonstrated comparable levels of microRNAs in serum and plasma,^36^ it is possible that some microRNAs might have been missed by analysing them only in plasma. Indeed, one of the microRNAs that we found significantly enriched in SMA serum compared to controls was miR‐206 (>40 fold) (Table S1), which has been previously identified as dysregulated in SMA animal models.^20^, ^37^, ^38^ However, in the therapeutic cohort study conducted in plasma, miR‐206 was barely detectable and hence was not included in the following analysis. Discrepant microRNA levels between serum and plasma have also been reported previously.^39^, ^40^ Another factor that could have influenced the findings could be due to the different methods used – while NGS was used in the discovery cohort study, a TaqMan qPCR assay was used in the therapeutic cohort study, and we cannot exclude different sensitivities in detecting miR‐206 between these two techniques.

Different sets of microRNAs were identified in this study when the two motor function assessment scales, CHOP‐INTEND and HINE, were analysed. The CHOP‐INTEND scale has been designed to assess motor function in weak infants and the rapid progression of the disease. HINE motor milestones scale could longitudinally monitor the development of motor milestones, such as sitting, head control, crawling or standing, which are milestones that some nusinersen‐treated SMA type 1 children can now acquire.^41^ Thus, the different aspects of these two assessment tools could lead to capturing different microRNAs that monitor the distinct functional abilities of the patients. Indeed, fewer microRNAs were found significant in the HINE scale analysis compared to the CHOP‐INTEND analysis. This is likely related to the fact that the CHOP‐INTEND measures more granular aspects of function, while the HINE concentrates on more gross functional improvements. Future studies on more longitudinal time points beyond 10 months of treatment may reveal more microRNAs able to indicate the response measured using the HINE scale.

Among the seven microRNAs whose baseline levels showed potential in predicting patients’ response to nusinersen treatment, miR‐378a‐3p was in common between both CHOP‐INTEND and HINE scales. Elevated levels of miR‐378a‐3p were associated with better motor function in nusinersen‐treated patients.

Pathway analysis of miR‐107, miR‐142‐5p, miR‐335‐5p, miR‐423‐3p, miR‐660‐5p, miR‐378a‐3p and miR‐23a‐3p identified three pathways involved in neurotrophin signalling (*p* = 0.0005,) mTOR signalling (*p* = 0.005) and FoxO signalling (*p* = 0.003) as enriched with genes targeted by the analysed microRNAs. These three pathways are all involved in the regulation of cell survival, autophagy and apoptosis.

miR‐107 has been reported to inhibit the apoptosis of myoblasts but also to negatively regulate myoblast differentiation by suppressing the expression of MyoD, MyoG and MyHC.^42^ A counteracting role was reported from miR‐378a‐3p that promotes skeletal muscle differentiation.^43^, ^44^ As a dynamic system, some myogenic cells will go through self‐renewal and return to quiescence, while others will proliferate and differentiate into myofibers, thus the two microRNAs likely reflect the different processes of skeletal muscle development.

A direct target of miR‐142‐5p is *FOXO3*, a transcriptional activator that induces the expression of autophagy genes, triggering proteolysis of skeletal muscles and activating apoptosis leading to neuronal cell death upon oxidative stress.^45^, ^46^ Overexpression of miR‐142‐5p significantly reduces the mRNA levels of *FOXO3* and enhances the expression of genes involved in skeletal muscle growth.^47^ In our study, we observed that higher baseline levels of miR‐142‐5p were associated with a better response to therapeutic intervention. We hypothesise that the increased levels of miR‐142‐5p, through suppression of *FOXO3*, may lead to reduced muscular atrophy and improved motor function acquirement.

Previously, miR‐335‐5p had been found to control self‐renewal or differentiation of mouse embryonic stem cells.^48^ Decreased level of miR‐335‐5p was detected in SMA‐induced pluripotent stem cells (iPSCs) compared to wild‐type iPSCs during the early steps of differentiation.^49^ In this study, we demonstrated that higher levels of miR‐335‐5p were associated with better motor function improvement in SMA patients. We also detected increased levels of miR‐335‐5p at 2 and 6 months after treatment. Our data may suggest a potential protective effect of miR‐335 in SMA.

Worth noting is miR‐23a‐3p, which we found increased in nusinersen patients with the higher motor ability both at baseline and following therapeutic intervention. MiR‐23a has been previously reported as having neuroprotective and skeletal muscle atrophy inhibiting properties^50^ and has been identified as a protective modifier of SMA.^51^ In the central nervous system, miR‐23a inhibits the expression of the apoptotic protease activating factor‐1 (Apaf‐1) and thus regulates the sensitivity of neurons to apoptosis.^50^ Overexpression of miR‐23a has also been shown to promote the expression of myelin genes through directly regulating the *PTEN* gene.^52^ In SMA iPSC‐derived motor neurons miR‐23a was found to be significantly reduced compared to iPSC‐derived motor neurons from healthy individuals.^51^ Transfection of synthetic miR‐23a into the SMA iPSC‐derived motor neurons led to reduced motor neuronal death, demonstrating that miR‐23a deficiency contributes to the pathology observed in SMA motor neurons.^51^ Furthermore, AAV9‐mediated expression of miR‐23a in the Smn^2B^/− SMA mouse model^53^ resulted in reduced neuromuscular junction defects, improved motor neuronal soma size, increased muscle fibre area and extended the survival of the Smn^2B^/− mice.^51^ In skeletal muscle, overexpression of miR‐23a downregulated *MAFbx/atrogin‐1* and *MuRF1* genes and provided resistance against atrophy exemplified by preserved muscle weight and cross‐sectional area of the muscles.^54^ These data indicate miR‐23a as an important microRNA to be characterised further in longer duration studies not only in patients receiving nusinersen but also in other SMA therapeutic interventions, including risdiplam and onasemnogene abeparvovec. In addition to representing a promising biomarker, this microRNA may also serve as an independent target for therapeutic intervention.

It is acknowledged that while the discovery cohort study included SMA type 2 and 3 patients, the therapeutic cohort only consisted of SMA type 1 patients who are more severe and younger than type 2 and type 3. In addition, while the median age in the discovery cohort was 10.2 years, the median age in the therapeutic cohort was only 19 months. The discrepancy in age and clinical severity between the two study cohorts might have resulted in some microRNAs specific to the severe type 1 patients being missed as the selection of microRNAs was based on data obtained in milder patients in the discovery cohort. Therefore, a further study in young and severe type 1 patients might be needed in the future to compliment our findings. Another limitation of this study is the relatively small number of patients and the short duration of follow‐up. While this is not uncommon for rare genetic diseases, our data need to be further validated in a larger cohort, and the actual added value of treatment response predictive value should be quantified after adjusting for age, disease duration, baseline CHOP‐INTEND and weight/height. Additionally, it will be interesting to investigate these microRNAs in patients treated by other disease‐modifying treatments such as risdiplam or onasemnogene abeparvovec.

In conclusion, we have identified a set of microRNAs in the blood of nusinersen‐treated SMA patients that could be considered as potential biomarkers to predict or monitor patients’ response to treatment. To further develop microRNAs in blood as biomarkers for SMA, more longitudinal studies in larger SMA cohorts will be needed to better understand the correlation of the microRNAs to disease progression and the improvement in motor function acquisition following different therapeutic interventions.

## Ethics Approval and Consent to Participate

This study has been approved by the Berkshire Research Ethics Committee (REC reference number 05/MRE12/32). All patients or legal guardians signed an informed consent form.

## Author Contributions

I. T. Z., L. S., F. M. and H. Z. conceived and prepared the manuscript. M. S. and K. A.‐G. contributed to the collection of clinical data. I. T. Z. and B. D. contributed to conducting the experiments. D. R. provided statistical advice. All authors read and approved the final draft.

## Conflict of Interest

F. Muntoni has received honoraria for presentation at meetings and scientific advisory boards of Biogen; Novartis and Roche. L. Servais, K. Aragon‐Gawinska received lecture honoraria and congress funding from Biogen and consultancy fees from Roche. No conflicts of interest from the other authors.

## Supporting information


**Figure S1.** Linear regression analysis between the age of start of nusinersen treatment and the relative expression at baseline of miR‐107.

**Table S1.** Differentially expressed microRNA in serum of SMA patients compared to controls.
**Table S2.** Differentially expressed microRNA (*p* < 0.05) in serum of SMA type 2 to SMA type 3 patients.
**Table S3.** Correlation between the baseline levels of microRNAs and the functional improvement of the patients at 2 and 6 months after treatment assessed using CHOP‐INTEND and HINE scales.
