# Supplementary figures and images for: Response of plasma microRNAs to nusinersen treatment in patients with SMA
Source: Ann Clin Transl Neurol. 2022 May 18;9(7):1011–26. doi: 10.1002/acn3.51579 (PMC9268869; doi:10.1002/acn3.51579)

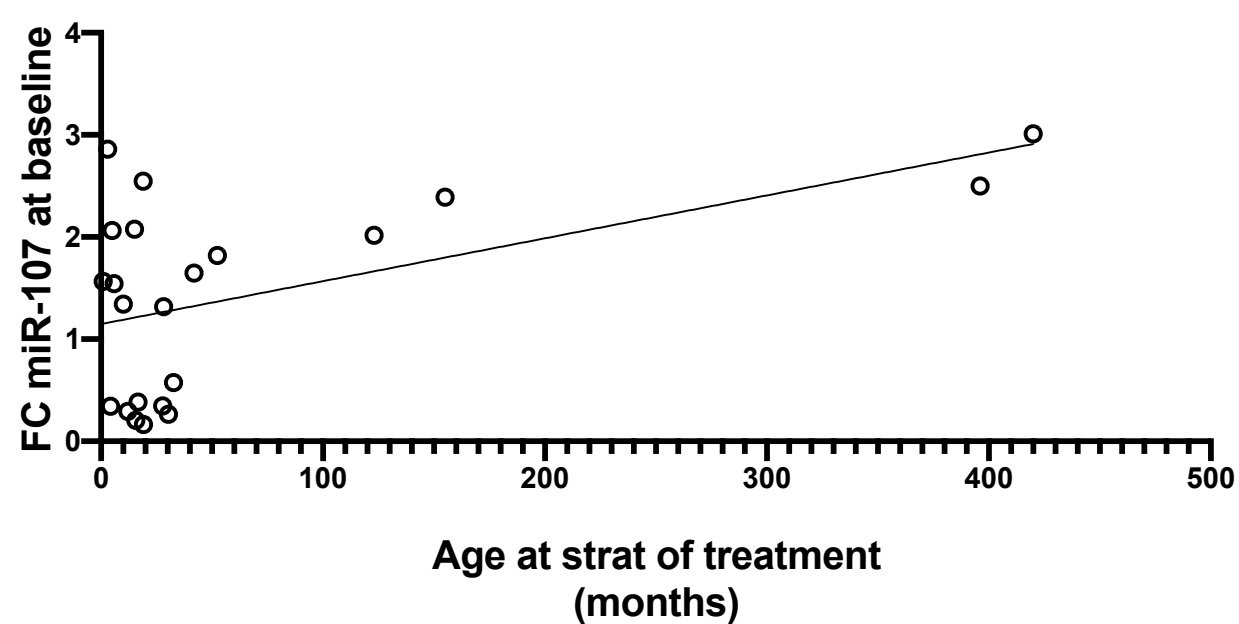

Supplement: Supplementary file 1 — Figure S1. Linear regression analysis between the age of start of nusinersen treatment and the relative expression at baseline of miR‐107. [file ACN3-9-1011-s001.pdf]
