# Supplementary material for: Response of plasma microRNAs to nusinersen treatment in patients with SMA
Source: Ann Clin Transl Neurol. 2022 May 18;9(7):1011–26. doi: 10.1002/acn3.51579 (PMC9268869; doi:10.1002/acn3.51579)
Supplement: Supplementary file 2 — Table S1. Differentially expressed microRNA in serum of SMA patients compared to controls. Table S2. Differentially expressed microRNA (p < 0.05) in serum of SMA type 2 to SMA type 3 patients. Table S3. Correlation between the baseline levels of microRNAs and the functional improvement of the patients at 2 and 6 months after treatment assessed using CHOP‐INTEND and HINE scales. [file ACN3-9-1011-s002.docx]

Supplementary tables

Table S1. Differentially expressed microRNA in serum of SMA patients compared to controls. The log fold change (logFC), uncorrected p-values, Benjamini-Hochberg FDR corrected p-values and the average normalised read counts in SMA and controls are presented.

Table S2. Differentially expressed microRNA (p-value < 0.05) in serum of SMA type 2 to SMA type 3 patients. The log fold change (logFC), uncorrected p-values, Benjamini-Hochberg FDR corrected p-values as well as the average normalised read counts in SMA type 2 and type 3 are presented.

Table S3: Correlation between the baseline levels of microRNAs and the functional improvement of the patients at 2 and 6 months after treatment assessed using CHOP-INTEND and HINE scales. Presented are the Spearman correlation coefficient and the *p*-values.

| microRNA | Response at 2 months CHOP-INTEND | | Response at 6 months CHOP-INTEND | | Response at 2 months HINE | | Response at 6 months HINE | |
| --- | --- | --- | --- | --- | --- | --- | --- | --- |
|  | Spearman coefficient | P-value | Spearman coefficient | P-value | Spearman coefficient | P-value | Spearman coefficient | P-value |
| let-7a-5p | 0.498 | 0.059 | 0.240 | 0.370 | 0.239 | 0.325 | -0.116 | 0.646 |
| let-7f-5p | **0.572** | **0.026** | 0.353 | 0.181 | 0.255 | 0.292 | 0.010 | 0.967 |
| miR-101-3p | 0.360 | 0.187 | 0.140 | 0.605 | 0.296 | 0.219 | -0.083 | 0.744 |
| miR-106a-5p | 0.416 | 0.123 | 0.261 | 0.329 | 0.231 | 0.341 | -0.131 | 0.605 |
| miR-106b-5p | 0.097 | 0.732 | 0.046 | 0.866 | 0.064 | 0.796 | -0.209 | 0.405 |
| miR-107 | **0.699** | **0.004** | **0.537** | **0.032** | 0.243 | 0.316 | -0.059 | 0.817 |
| miR-126-5p | -0.181 | 0.519 | -0.171 | 0.526 | 0.239 | 0.323 | 0.146 | 0.562 |
| miR-139-5p | -0.179 | 0.523 | -0.118 | 0.663 | 0.364 | 0.126 | **0.473** | **0.048** |
| miR-142-5p | **0.663** | **0.007** | 0.478 | 0.061 | 0.374 | 0.115 | 0.204 | 0.417 |
| miR-144-3p | 0.441 | 0.100 | 0.252 | 0.346 | 0.296 | 0.219 | 0.013 | 0.961 |
| miR-144-5p | 0.208 | 0.457 | 0.131 | 0.628 | 0.127 | 0.604 | -0.220 | 0.381 |
| miR-15b-3p | 0.226 | 0.418 | 0.099 | 0.716 | 0.280 | 0.246 | -0.082 | 0.748 |
| miR-16-5p | -0.412 | 0.127 | -0.313 | 0.238 | -0.121 | 0.623 | -0.106 | 0.676 |
| miR-17-5p | 0.484 | 0.068 | 0.308 | 0.245 | 0.271 | 0.262 | -0.126 | 0.620 |
| miR-181b-5p | 0.416 | 0.123 | 0.193 | 0.473 | 0.361 | 0.129 | -0.081 | 0.751 |
| miR-186-5p | 0.339 | 0.217 | 0.257 | 0.337 | 0.327 | 0.172 | -0.089 | 0.726 |
| miR-19b-3p | 0.430 | 0.110 | 0.211 | 0.433 | **0.465** | **0.045** | 0.087 | 0.732 |
| miR-20a-5p | 0.357 | 0.192 | 0.168 | 0.534 | 0.249 | 0.305 | -0.157 | 0.534 |
| miR-20b-5p | 0.287 | 0.320 | 0.139 | 0.609 | 0.155 | 0.539 | -0.211 | 0.400 |
| miR-30b-5p | 0.116 | 0.679 | 0.034 | 0.901 | 0.312 | 0.193 | 0.287 | 0.249 |
| miR-30e-5p | **0.520** | **0.047** | 0.357 | 0.175 | 0.410 | 0.081 | 0.016 | 0.951 |
| miR-32-5p | 0.403 | 0.136 | 0.255 | 0.340 | 0.286 | 0.236 | 0.062 | 0.808 |
| miR-328-3p | 0.493 | 0.062 | 0.468 | 0.068 | 0.197 | 0.419 | 0.219 | 0.383 |
| miR-335-5p | **0.590** | **0.021** | **0.503** | **0.047** | **0.481** | **0.037** | 0.263 | 0.292 |
| miR-340-5p | **0.554** | **0.032** | 0.457 | 0.075 | 0.370 | 0.119 | 0.338 | 0.170 |
| miR-363-3p | 0.475 | 0.074 | 0.273 | 0.307 | 0.374 | 0.115 | 0.044 | 0.863 |
| miR-378a-3p | 0.267 | 0.336 | 0.004 | 0.987 | **0.585** | **0.009** | 0.294 | 0.236 |
| miR-423-3p | **0.613** | **0.015** | 0.444 | 0.085 | 0.292 | 0.225 | 0.190 | 0.449 |
| miR-432-5p | -0.208 | 0.457 | -0.062 | 0.820 | 0.071 | 0.773 | 0.246 | 0.325 |
| miR-451a | 0.384 | 0.158 | 0.196 | 0.467 | 0.232 | 0.339 | -0.052 | 0.837 |
| miR-532-5p | 0.013 | 0.965 | -0.109 | 0.687 | 0.335 | 0.161 | -0.021 | 0.934 |
| miR-584-5p | -0.168 | 0.583 | -0.020 | 0.944 | -0.224 | 0.387 | 0.050 | 0.850 |
| miR-660-5p | **0.599** | **0.018** | 0.401 | 0.124 | 0.388 | 0.101 | 0.032 | 0.898 |
| Y_RNA.122-201_1 | 0.513 | 0.051 | 0.240 | 0.370 | 0.270 | 0.264 | 0.009 | 0.970 |
| let-7e-5p | 0.443 | 0.098 | 0.383 | 0.143 | 0.176 | 0.471 | 0.005 | 0.984 |
| miR-93-5p | 0.464 | 0.081 | 0.201 | 0.456 | 0.290 | 0.228 | 0.000 | 1.000 |
| miR-125a-5p | 0.464 | 0.081 | 0.338 | 0.201 | 0.301 | 0.210 | 0.049 | 0.846 |
| miR-194-5p | -0.050 | 0.859 | -0.146 | 0.589 | 0.285 | 0.238 | 0.109 | 0.667 |
| miR-26b-5p | 0.011 | 0.970 | -0.124 | 0.648 | 0.093 | 0.705 | -0.184 | 0.465 |
| miR-99b-5p | -0.057 | 0.846 | -0.233 | 0.404 | 0.103 | 0.683 | 0.195 | 0.454 |
| miR-23a-3p | 0.294 | 0.288 | 0.094 | 0.728 | **0.578** | **0.009** | **0.542** | **0.020** |
